# Incidence and antimicrobial susceptibility of *Neisseria gonorrhoeae* isolates from patients attending the national *Neisseria gonorrhoeae*reference laboratory of Hungary

**DOI:** 10.1186/1471-2334-14-433

**Published:** 2014-08-06

**Authors:** Alexandra Brunner, Eva Nemes-Nikodem, Noemi Mihalik, Marta Marschalko, Sarolta Karpati, Eszter Ostorhazi

**Affiliations:** Department of Dermatology, Venerology and Dermatooncology, Semmelweis University, Mária u. 41, 1085 Budapest, Hungary; Institute of Medical Microbiology, Semmelweis University, Nagyvárad tér 4, 1089 Budapest, Hungary

**Keywords:** *Neisseria gonorrhoeae*, Antimicrobial resistance, Incidence, MIC, Hungary

## Abstract

**Background:**

The Hungarian national guidelines for the treatment of gonorrhoea were published in 2002 but are now widely considered to be outdated. Improved knowledge is needed with respect to the epidemiology and antimicrobial susceptibility of *Neisseria gonorrhoeae* strains currently circulating in Hungary not least for the construction of updated local recommendations for treating gonorrhoea. European guidelines are based mostly on western European data raising concerns locally that recommended treatments might not be optimised for the situation in Hungary. We report our recent study on the distribution of antibiotic resistance in various Hungarian (East European) *Neisseria gonorrhoeae* strains isolated from patients with gonorrhoea over the past four years.

**Methods:**

Between January 2010 and December 2013, isolates of *N. gonorrhoeae* were obtained from sexually active individuals during medical examination at the STD Center of Semmelweis University in Budapest. The minimal inhibitory concentrations (MIC) of azithromycin, cefixime, ceftriaxone, ciprofloxacin, penicillin, tetracycline and spectinomycin were determined to establish the antimicrobial susceptibility of the strains currently circulating in patients that attend our clinic.

**Results:**

Among the 9097 patients tested, 582 had an *N. gonorrhoeae* infection as detected by culture. The isolates were all sensitive to ceftriaxone and spectinomycin and 581/582 strains were sensitive to cefixime. In contrast, the number of detected strains with elevated azithromycin MIC did increase over the time period examined to approximately 16% in 2013. There was a high percentage of detected resistance to penicillin (77%), tetracycline (86%), and ciprofloxacin (66%) in the isolates examined in this study.

**Conclusion:**

Current European guidelines recommend 2 g azithromycin in addition to 500 mg ceftriaxone as first choice therapy for gonorrhoea. For the purposes of revising the Hungarian national treatment guidelines, apparent increasing resistance to azithromycin during the last four years should be accounted for. It is also clear that penicillin, tetracycline and ciprofloxacin are inappropriate treatment measures at least locally. We also recommend that culture should form part of the diagnostic pathway of gonorrhoea, followed by antibiotic susceptibility testing with MIC determination. This will provide valuable continued monitoring of antibiotic resistance development in strains of *Neisseria gonorrhoeae* circulating in Hungary.

**Electronic supplementary material:**

The online version of this article (doi:10.1186/1471-2334-14-433) contains supplementary material, which is available to authorized users.

## Background

*N. gonorrhoeae* infections represent 106 million of the estimated 498 million new cases of curable sexually transmitted infection (STI) that occur globally every year [1]. In 2008, the World Health Organization (WHO) estimated 3.4 million cases among adults in the European Union (EU) [1]. Gonorrhoea remains the second most commonly reported bacterial STI after chlamydia infection [2]. Gonococcal infection, depending on the anatomic site of exposure can cause acute urethritis, cervicitis, proctitis or pharyngitis. However, most cervical, pharyngeal and rectal infections are asymptomatic. Untreated or inadequately treated gonorrhoea can cause serious reproductive complications in women. This includes pelvic inflammatory disease, infertility and ectopic pregnancy.

Hungary has a population of 10 million people and is served by 135 sexual health care units that are financed by the government. There are also more than 300 private venerologists available to help patients with STDs. In Hungary, gonorrhoeal infection cases must be notified to the National Epidemiological Center regardless of whether they were detected with nucleic acid amplification tests (NAATs), culture or only microscopically. The notification contains the age, gender and sexual orientation of the patient, the anatomical sites of the infection and the region of the country where the infection was detected.

The Hungarian national guidelines on the diagnosis and treatment of gonorrhoea [3] were published in 2002. These guidelines recommend 1x 250 mg ceftriaxone i.m. or 400 mg ofloxacin per os as first choice treatment, or 400 mg cefixime per os or 500 mg ciprofloxacin per os can be administered in cases of uncomplicated gonorrhoeal urethritis or cervicitis. The guidelines also allow diagnosis of gonorrhoeal urethritis in symptomatic men based only on a Gram stain of the male urethral specimen and a demonstration of the presence of polymorphonuclear leukocytes with intracellular Gram-negative diplococci. While this test is fast to administer and typically very reliable, it limits any opportunities to monitor the development of antibiotic resistance in *N. gonorrhoeae* – a phenomenon that is causing widespread concern globally.

In 2013, *N. gonorrhoeae* infections were notified in 1563 cases in Hungary [4]. However no reliable data with respect to antimicrobial resistance (AMR) exists because most diagnoses were based on microscopic analysis or NAATs and did not include any culturing (which is required for determining AMR). The National Reference Laboratory of *N. gonorrhoeae*, a part of the STD Center of Semmelweis University, receives patients and samples from all over the country. At our centre, diagnosis of gonorrhoea is made by culture, meaning that AMR development within Hungary can be monitored retrospectively.

The aims of this study were therefore to investigate *N. gonorrhoeae* infection rates according to gender, age and anatomic location of the symptoms, and to determine phenotypic AMR of the isolates obtained from patients with gonorrhoea who visited our clinic over the previous four years. A specific objective of the study was to understand the appropriateness of current Hungarian treatment recommendations especially with respect to suspected local development of AMR in circulating *N. gonorrhoreae* strains.

## Methods

All studies were carried out at the STD Center of Department of Dermatology, Venereology and Dermatooncology of Semmelweis University, Budapest, Hungary. Over a four-year period (January 2010 to December 2013), *N. gonorrhoeae* isolates were obtained from patients attending medical examinations for a suspected STI. During medical examination by a physician, patient data was also collected with respect to their age, sex, sexual orientation and anatomic site of infection. The isolates were cultured from consecutively symptomatic gonorrhoea patients and from their asymptomatic contacts. All isolates were collected as part of standard patient care and patients gave and signed informed consent. Patient data was analysed according to law 1997/CLIV 26§ taking into account maximum privacy rights and anonimity of patients [5]. Ethical approval signed by the Semmelweis University Regional and Institutional Committee of Science and Research Ethics was not required for the study because all used methods were not more than normally needed for diagnosis and treatment.

For the identification of *N. gonorrhoeae,* cervical, anal, urethral and pharyngeal swabs were taken and cultured on preheated VCA3 agar (Biomérieux, Budapest, Hungary) and on non-selective PVX chocolate agar (Biomérieux, Budapest, Hungary). Cultures were incubated for 24-48 hours at a temperature of 36.5°C with 5% carbon dioxide, and their identification and penicillinase production was confirmed using NH-API strip tests (Biomérieux, Budapest, Hungary).

Minimum inhibitory concentrations (MIC; mg/L) of cefixime, ceftriaxone, penicillin, tetracycline, azithromycin, spectinomycin and ciprofloxacin were determined on PVX chocolate agar (Biomérieux, Budapest, Hungary) using MIC strip tests (Liofilchem® s.r.l., Roseto degli Abruzzi, Italy), according to the manufacturer’s instructions, and by using a direct colony suspension equivalent to a 0.5 McFarland standard. Testing conditions also included incubation at 36.5°C and 5% carbon dioxide for 24 hours. All results were interpreted by using breakpoints for susceptibility and resistance according to the European Committee on Antimicrobial Susceptibility Testing (EUCAST) (Table 1) [6]. To ensure the quality of the culture media and susceptibility tests, *N. gonorrhoeae* ATCC 49226 was used as a control strain. Isolates were classified as having a presumed high level of resistance to tetracycline (TRNG) if MIC was ≥16 mg/L, and as having a presumed plasmid-mediated resistance to penicillin (PPNG) if they were ß-lactamase positive [7].

**Table 1:** ***N. gonorrhoeae*** **MIC breakpoints for susceptibility and resistance according to the European Committee on Antimicrobial Susceptibility Testing (EUCAST)**

	Sensitive (mg/L)	Resistant (mg/L)
penicillin	≤ 0,06	> 1
cefixime	≤ 0,12	> 0,12
ceftriaxone	≤ 0,12	> 0,12
ciprofloxacin	≤ 0,03	> 0,06
azithromycin	≤ 0,25	> 0,5
tetracycline	≤ 0,5	> 1
spectinomycin	≤ 64	> 64

Source: [http://www.who.int/drugresistance/Antimicrobial_resistance_in_Neisseria_gonorrhoeae.pdf].

## Results

We examined the prevalence of *N. gonorrhoeae* infection in 9097 sexually active patients attending the Hungarian National STD Center between January 2010 and December 2013. A total of 26383 swab samples were collected during the study period. These samples, taken from urethra, cervix, anus, pharynx or conjunctiva were examined by culture. Over the period of the four years of the study the number of patients who visited the STD Center increased by 10.0% (Table 2). Depending on the symptoms observed, gender and anamnesis, one to five swab samples were collected for culturing. There was a concurrent increase in the number of samples collected of 8.7% over the study period (Table 2). The ratio of *N. gonorrhoeae* positive samples to total samples collected increased from 1.9% at the beginning of 2010 to 3.2% by the end of 2013. The ratio of positive patients to total patients examined increased from 5.2% in 2010 to 7.8% by the end of 2013 (Table 2). Out of the total number of samples collected, *N. gonorrhoeae* infection was confirmed in 673 samples and 582 patients (of both sexes). This included 16 samples taken from five patients who had returned to the clinic with new infections.

**Table 2:** **Number of patients, samples for culture,** ***N. gonorrhoeae*** **positive samples and** ***N. gonorrhoeae*** **infected patients at the STD Centre of Semmelweis University, 2010-2013**

	2010	2011	2012	2013	Total
Number of patients	2167	2229	2317	2384	9097
Number of tested samples	6397	6350	6682	6954	26383
*N. gonorrhoeae* positive samples (Number/%)	129/1.9%	144/2.2%	178/2.7%	222/3.2%	673/2.5%
*N. gonorrhoeae* infected patients (Number/%)	112/5.2%	124/5.6%	159/6.9%	187/7.8%	582/6.4%

**Figure 1:**
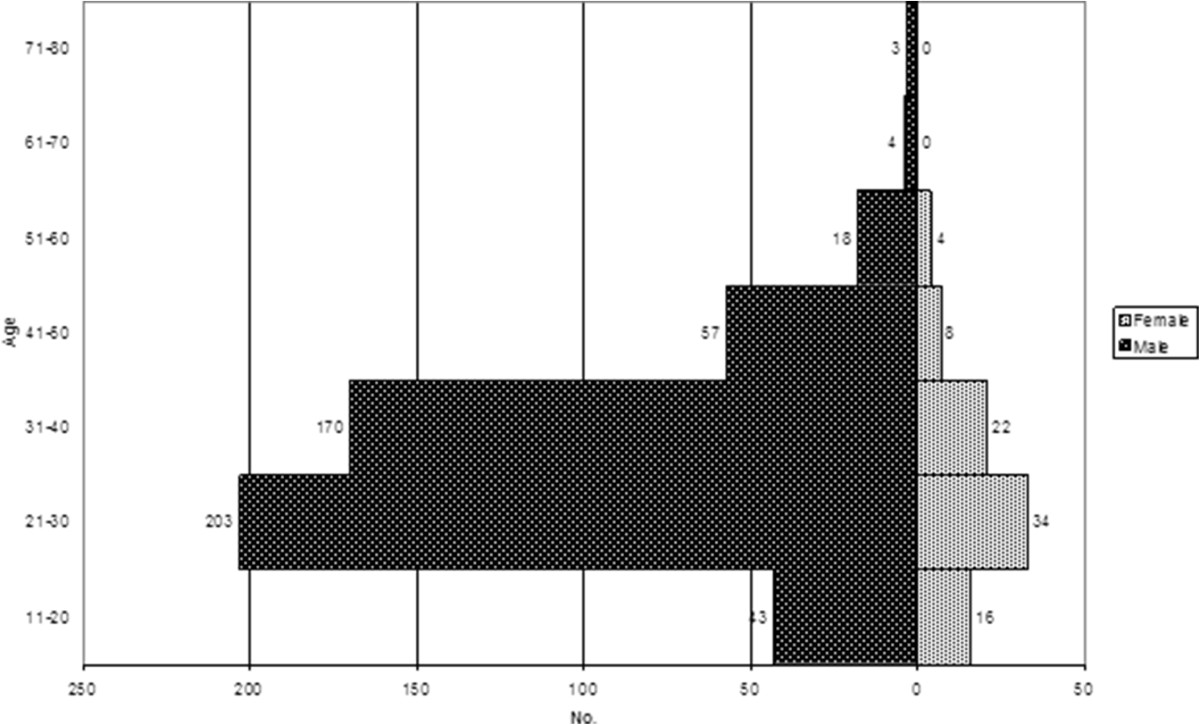
**Distribution of** ***N. gonorrhoeae*** **positive patients according to their age at the time of infection between January 2010 and December 2013.**

In terms of total population studied the majority of patients attending the clinic were male in all years of the study. Specifically, the male to female ratio of patients attending the STD Center were 62/38%, 58/42%, 57/43% and 61/39% in 2010, 2011, 2012 and 2013 respectively. On average over the study period 86% (498/582) of cases examined that were subsequently determined to be positive for *N. gonorrhoeae* were men, with the balance of cases (14% (84/582)) female. 86% (96/112), 85.5% (106/144), 84% (133/159) and 87% (163/187) were male patients in 2010, 2011, 2012 and 2013, respectively. Ages ranged from 14 to 76 years with an average of 31.7 years for male and 26.7 years for female patients (Figure 1). Among the infected male patients, 19% (95/498) declared that they were men who have sex with men (MSM), while 81% (403/495) declared they were heterosexual. The dominant anatomical site of gonococcal infections was urethra in male (80%, 437/548) and cervix in female patients (62%, 77/125). Pharyngeal and anal infections were identified both separately and in parallel with the dominant urethral infection in men or cervical infection in female patients. Only one extragenital *N. gonorrhoeae* infection causing conjunctivitis was detected in a male patient, who had gonorrhoeal urethritis and pharyngitis at the same time (Table 3).

**Table 3:** **Distribution of specimens cultured for** ***N. gonorrhoeae*** **strains between January 2010 and December 2013**

Samples	Men	%	Women	%
Cervix	0	0	77	61.6
Pharynx	22	4.0	11	8.8
Anus	88	16.1	10	8.0
Urethra	437	79.7	27	21.6
Conjunctiva	1	0.2	0	0
Total	548	100	125	100

We performed antimicrobial susceptibility tests on all 673 isolates. We found that isolates from different anatomical sites of the same patients did not differ in terms of MICs for the antibiotics we tested. Based on this observation, calculated percentages are based on data from single isolates (as in one strain) per patient (582 samples in total). The antimicrobial susceptibility rates of *N. gonorrhoeae* to azithromycin, cefixime, ceftriaxone, ciprofloxacin, penicillin, tetracycline and spectinomycin are given in Table 4. Almost all isolates were susceptible to ceftriaxone, cefixime (MIC ≤ 0.12 mg/L) and spectinomycin (MIC ≤ 64 mg/L). However there were small increases in the MIC averages for ceftriaxone, cefixime and spectinomycin between the beginning of 2010 and the end of 2013. These were 0.002 mg/L to 0.019 mg/L for ceftriaxone, 0.016 mg/L to 0.047 mg/L for cefixime and 2 mg/L to 8 mg/L for spectinomycin. In the last case, we did identify two isolates with an MIC of 48 mg/L. In 2013 we also observed for the first time (in our laboratory) three strains that exceeded breakpoints: two strains with a cefixime MIC of 0.125 mg/L and one strain with a cefixime MIC of 0.19 mg/L. In strict accordance with EUCAST recommendations this means 0.51% of the strains had a cefixime MIC just above the resistant breakpoint. Resistance to azithromycin (MIC > 0.5 mg/L) increased from 0 to 15.86% of strains tested during the four years of the study.

**Table 4:** **Distribution of antibiotic resistant** ***N. gonorrhoeae*** **strains (%) in Hungary**

Resistance to antibiotics/year	2010	2011	2012	2013
Azithromycin	0	0.8	6.9	15.9
Cefixime	0	0	0	0.2
Ceftriaxone	0	0	0	0
Ciprofloxacin	70.5	61.7	64.6	67.2
Penicillin	75.4	76.2	77.3	79.5
Spectinomycin	0	0	0	0
Tetracycline	88.1	87.5	82.9	85.5

The prevalence of ciprofloxacin and tetracycline resistance varied year to year but was consistently high for both antibiotics (66% and 86%, respectively) (see Table 4). The prevalence of penicillin resistance appeared to slowly increase from 75 to 79% in the four years of the study. PPNG was detected in 16.2% of the isolates and TRNG in 23%, whereas 14.8% of the isolates presented both phenotypes (PPNG/TRNG).

## Discussion

In Hungary, as is the case in many other countries, the control of gonococcal infections mostly relies on an effective single-dose antibiotic therapy given at the first clinical presentation of the patient. Prior knowledge of the antimicrobial susceptibility pattern of the particular strain infecting the patient is routinely not established however. With increasing evidence of antimicrobial resistance in gonococcal infections there is therefore a real risk of treatment failure and development of further resistance to antibiotics. Current Hungarian guidelines for the diagnosis and treatment of gonorrhoea date from 2002 and are based on bygone national and international data of gonococcal resistance. Our study therefore sought to understand ongoing resistance trends with respect to gonorrhoea in Hungary, and to ultimately contribute to the preparation of new guidelines to ensure the success of any therapies targeting gonorrhoea. Our data, based on sampling over the previous four years highlight that there appears to be continued antimicrobial resistance in circulating strains of *N. gonorrhoeae* strains to a variety of common antibiotics (including high rates of resistance to ciprofloxacin, which is one of the antibiotics recommended in the national Hungarian guidelines) and that there may be developing resistance to others (including azithromycin and penicillin and possibly even cefixime) in this part of eastern Europe.

Over the last 70–80 years treatment options have diminished due to the emergence and spread of resistance to all drugs recommended for treatment of gonorrhoea. Plasmid-mediated resistance mechanisms have been described for penicillins (PPNG) and tetracyclines (TRNG), but not for sulphonamides, macrolides, spectinomycin, aminoglycosides, quinolones and cephalosporines. In these cases only chromosomal mutations have been described as the reason for resistance [8]. Since *N. gonorrhoeae* is naturally competent for the uptake and recombination of external DNA during its entire life cycle, the transfer of chromosomally encoded resistance genes is both rapid and extensive in comparison to uptake of plasmid-mediated resistance genes [9]. Resistance may initially emerge in the commensal *Neisseria spp*. inhabiting the human body as these are exposed more frequently to antimicrobials than the transiently acquired gonococci. The commensal *Neisseria spp.* can act as a reservoir of resistance genes that can be transferred to gonococci through transformation. In our study *N. gonorrhoeae* was detected in pharyngeal swab samples of symptomatic or asymptomatic infected patients in 4 and 8% of male and female patients, respectively. This may be significant as the pharynx may be an ideal anatomic site to provide the opportunity for chromosomally encoded resistance development. Pharyngeal gonorrhoea is mostly asymptomatic meaning that commensal *Neisseria spp*. and *N. gonorrhoeae* can co-exist for extended time periods in the pharynx and share genetic material [10–12]. This is likely to include the transfer of antibiotic resistance genes and therefore the development of antibiotic resistance in *N. gonorrhoeae.* As humans appear to be the obligate host of *N. gonorrhoeae* it would appear that control of resistance lies in appropriate treatment regimes and that includes correct usage of antibiotics. The emergence and spread of antimicrobial resistance in *N. gonorrhoeae* all over the world requires the control of incidence of gonorrhoea through education and appropriate sexual practices and control of antimicrobial resistance through appropriate antibiotic usage. This is particularly the case in Hungary where both incidence of gonorrhoea and antibiotic resistance appear to be increasing in recent years.

According to data from the National Epidemiological Center the incidence of *N. gonorrhoeae* infection appears to be increasing in Hungary - the number of the reported (notified) cases was 1158 in 2010, 1369 in 2011, 1491 in 2012 and 1563 in 2013 [4]. In the report of the National Epidemiological Center, 75-82% of the infections were detected in male patients and the dominant site of infection was urethra in male (81%), and cervix in female (62%) (in this time period in Hungary). About 10% of the notifiable infections were diagnosed at the STD Center of Semmelweis University during this period, and the distribution according to gender, age and location of the symptoms was the same as the national results.

To our knowledge the only available data relating to the distribution of resistance against different antibiotics of the Hungarian *N. gonorrhoeae* strains exist in the surveillance reports of EURO-GASP (European Gonococcal Antimicrobial Surveillance Programme) [13, 14]. In these projects 14 and 13 *N. gonorrhoeae* strains were collected from Hungary in 2010 and 2011. These data were collected from a sentinel laboratory based on the report of a selected group of physicians [13, 14]. In these reports there were no Hungarian *N. gonorrhoeae* strains with PPNG property. All these strains were susceptible to azithromycin, and 79% or 61.5% were resistant to ciprofloxacin in 2010 and 2011, respectively. These data contained no occurrence of cefixime resistant strains in 2010, but 1% of the isolates had decreased susceptibility to cefixime in 2011.

In our work we processed 582 *N. gonorrhoeae* strains, which corresponds to approximately 10% of the total number of notified infections in Hungary during the period between the beginning of 2010 and the end of 2013. This provides a more representative assessment of antimicrobial resistance to *N. gonorrhoeae* than any other previous survey carried out in Hungary.

Among our 582 *N. gonorrhoeae* isolates generally no resistance occurred against ceftriaxone, cefixime and spectinomycin. These results were interpreted using breakpoints for susceptibility and resistance according to the EUCAST [6]. All the measured MICs were not higher than their respective resistance breakpoints, with the exception of three strains isolated in 2013, which showed slightly raised resistance towards cefixime. Year on year there was a small increase in MIC averages for ceftriaxone, cefixime and spectinomycin. These averages did not exceed resistance breakpoints with the exception of the three cases mentioned above. We also found a small increase in the number of penicillin resistant strains during the study period. The rate of PPNG strains was stable at around 16%, which suggests different chromosomal mutations may be reasonably assumed for this type of resistance development. The acquisition of a mosaic *penA* gene encoding a remodelled penicillin binding protein (PBP2) and overproduction of an efflux pump in *N. gonorrhoeae* appears to be responsible for reduced susceptibility to cephalosporins [15]. Since the first ceftriaxone-resistant isolates were identified in Japan (2009), France (2010), and Spain (2011) [16–18], a real threat now exists that extensively resistant strains of *N. gonorrhoeae* may emerge and spread worldwide. Enhanced surveillance for resistance against other anti-gonococcal agents is therefore warranted.

According to the EUCAST interpretations all of the strains were susceptible to spectinomycin with a MIC lower than 64 mg/L. The average spectinomycin MIC increased during the study period and we did identify two isolates with clearly elevated (48 mg/L) MICs, suggesting that only intermediate sensitivity can be assured (according to the recommendation of Clinical and Laboratory Standards Institute (CLSI) [19]). Verified resistance to spectinomycin is exceedingly rare worldwide with only five spectinomycin-resistant isolates (all between 1988 and 1990) identified in the US Gonococcal Isolate Surveillance Project [20] using the CLSI recommendation. In comparison no spectinomycin-resistant isolates have been identified in the EURO-GASP in 2010-2011 [13, 14].

The very common occurrence (82-88% in Hungary over the past four years in our study) of tetracycline resistant strains is not unique in comparison to other countries. Despite some exceptions (in the USA, tetracycline resistance rates are only 22.5% [21] while rates in South America and the Caribbean decreased from a level of 61.1% of isolates tested in 2001 to 21.8% in 2010 [22]), high prevalence of tetracycline resistance has been detected in some countries in South East Asia. Bhutan and Indonesia reported a resistance rate of more than 95% between 2009 and 2012 [23], and a rate of 92.8% tetracycline resistance was noted in North Africa in 2009 [24]. This high percentage of detected resistance to tetracycline indicates that this antibiotic is certainly not appropriate for gonorrhoea treatment in Hungary and probably elsewhere.

Furthermore, the effectiveness of the quinolone antibiotics appears to be equally limited. For example, the prevalence of ciprofloxacin resistant strains that we detected in this study was between 61-70% over the previous four years. In comparison, prevalence of ciprofloxacin resistance in the USA was 13.5% [21], while in Latin America and the Caribbean ciprofloxacin rates increased from 1.6% of isolates tested in 1997 to 42.1% in 2010 [21]. Rates of ciprofloxacin resistance have decreased continuously since 2009 in the participant countries of EURO-GASP, but still remained as high as 52.7% and 48.7% in 2010 and 2011, respectively [13, 14]. The usage of ciprofloxacin, which was previously recommended in gonorrhoea treatment in Hungary, is no longer suitable according to the rates of resistance we observed in recent years.

Resistance to azithromycin (MIC > 0.5 mg/L) increased from 0 to 15.86% during the four years of our study. The Centers for Disease Control and Prevention [25] and the International Union against Sexually Transmitted Infections (IUSTI) [26] published global and European guidelines for diagnosis and/or treatment of *N. gonorrhoeae* to combat and mitigate the spread of multidrug-resistant gonorrhoea. Their new treatment guidelines revised the recommended treatment to be 250 mg or 500 mg ceftriaxone im combined with 1 g or 2 g azithromycin per os, respectively. However, based on past and current data reported in this study, *N. gonorrhoeae* may continue to emerge with azithromycin resistance in our country, especially as increased usage of this antibiotic is expected. The World Health Organization has suggested that an antimicrobial should not be used when >5% of strains demonstrate resistance [27]. If our data can be verified it would appear that this cut-off has already been passed in Hungary. In addition to complying with the new recommendations it is necessary to also adequately run antimicrobial surveillance programmes. It would be advisable to now include surveillance for ceftriaxone and azithromycin resistance, not least to control the emergence of extremely drug resistant strains.

## Conclusion

Our study contributes large amounts of data on the current antimicrobial susceptibility rates of circulating *N. gonorrhoeae* strains in Hungary in the period between the beginning of 2010 and up to the end of 2013. It is clear that there are considerable levels of antimicrobial resistance in circulating strains and in some cases resistance may be emerging. Of concern is that such resistance may be emerging towards antibiotics that are currently recommended as first line treatments for gonorrhoea in Hungary. Current diagnosis approaches typically only utilise Gram staining techniques, which while regarded as highly accurate, do not allow antibiotic resistance to be monitored routinely. Based on our observations we are now of the opinion that culture and susceptibility testing are indispensable in the currently developing situation of multidrug resistance among the *N. gonorrhoeae* strains. It is somewhat reassuring that during the previous four years, resistance has not been identified towards the recommended treatment of ceftriaxone in Hungary. However, in Europe extensively drug resistant *N. gonorrhoeae* strains have emerged and we believe it is only a matter of time before this occurs in Hungary if appropriate control measures and surveillance are not implemented soon. This emergence of resistance should prompt revision of our national diagnosis and treatment guidelines. The recommendations of IUSTI and other international organisations involved in developing advice and treatment approaches for gonorrhoea should be considered. However in Hungary the incidence of azithromycin resistance appears to have increased over recent years and this should be taken into account in developing any new guidelines.

## Authors’ original submitted files for images

Below are the links to the authors’ original submitted files for images. Authors’ original file for figure 1 Authors’ original file for figure 2

## Abbreviations

AMR: Antimicrobial resistance
CLSI: Clinical and laboratory standards institute
EUCAST: European committee on antimicrobial susceptibility testing
EURO-GASP: European gonococcal antimicrobial surveillance programme
IUSTI: International union against sexually transmitted infections
MIC: Minimal inhibitory concentrations
MSM: Men who have sex with men
PBP: Penicillin binding protein
PPNG: Penicillinase producing *Neisseria gonorrhoeae*
TRNG: Tetracycline resistant *Neisseria gonorrhoeae*.
