# Supplementary figures and images for: Incidence and antimicrobial susceptibility of Neisseria gonorrhoeae isolates from patients attending the national Neisseria gonorrhoeaereference laboratory of Hungary
Source: BMC Infect Dis. 2014 Aug 6;14:433. doi: 10.1186/1471-2334-14-433 (PMC4155111; doi:10.1186/1471-2334-14-433)

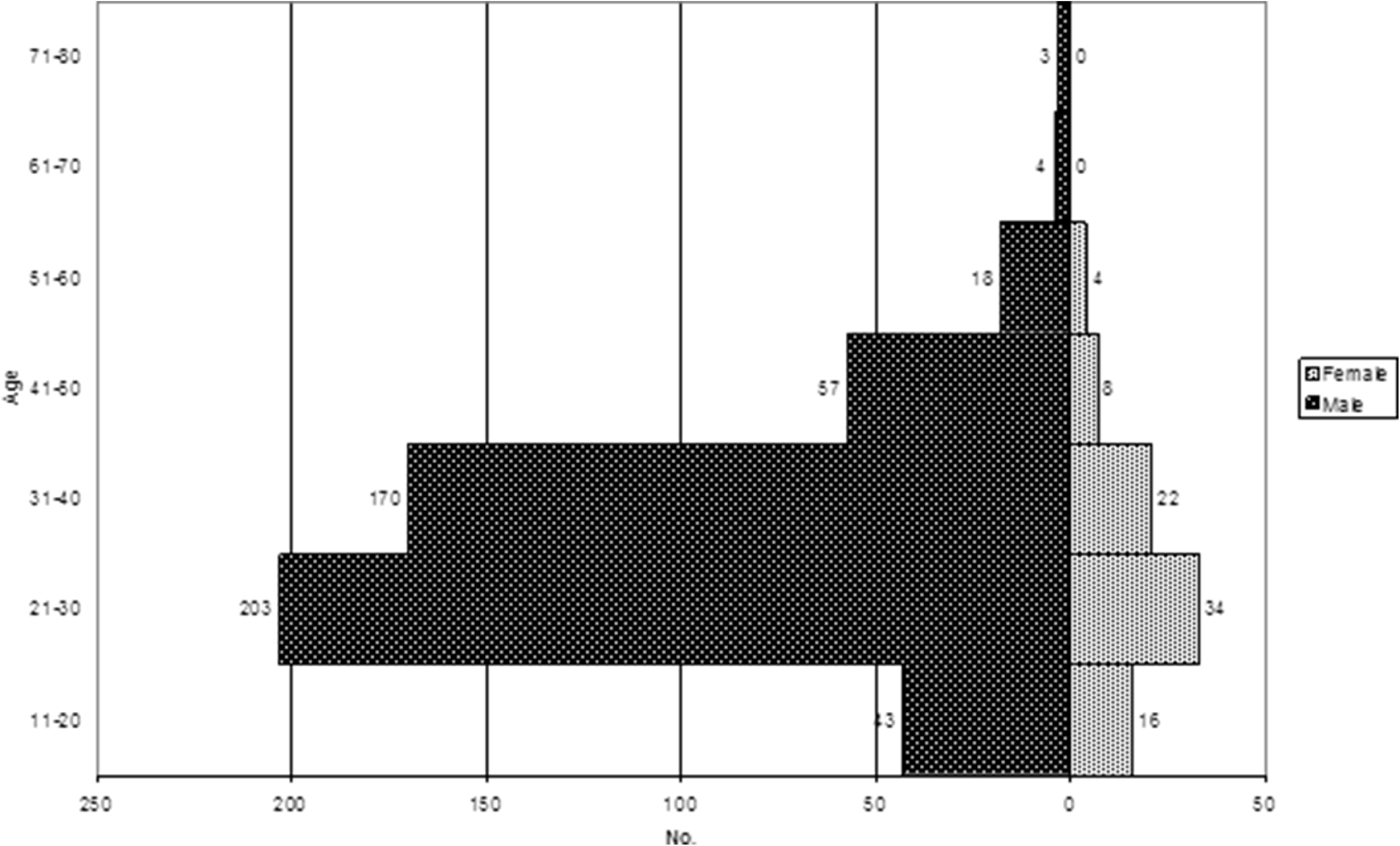

Supplement: Supplementary file 1 — Authors’ original file for figure 1 [file 12879_2014_3757_MOESM1_ESM.tiff]

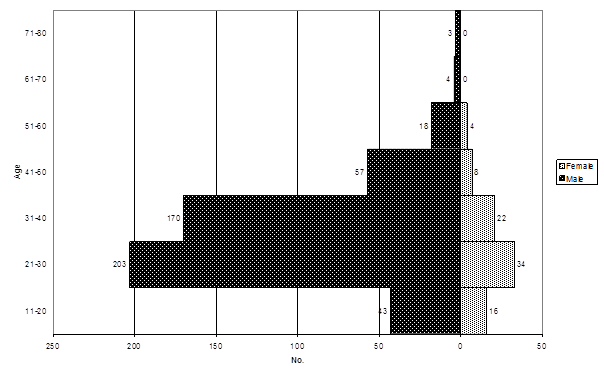

Supplement: Supplementary file 2 — Authors’ original file for figure 2 [file 12879_2014_3757_MOESM2_ESM.tiff]
